# An Institutional Retrospective Analysis of 93 Patients with Brain Metastases from Breast Cancer: Treatment Outcomes, Diagnosis-Specific Prognostic Factors

**DOI:** 10.3390/ijms131216489

**Published:** 2012-12-05

**Authors:** Delphine Antoni, Jean-Baptiste Clavier, Marius Pop, Céline Benoît, François Lefebvre, Georges Noël

**Affiliations:** 1 Department of Radiation Oncology, Centre de lutte Contre le Cancer Paul Strauss, 3, rue de la Porte de l’hôpital, 67065 Strasbourg cedex, France; E-Mails: jclavier@strasbourg.unicancer.fr (J.-B.C.); mpop@strasbourg.unicancer.fr (M.P.); gnoel@strasbourg.unicancer.fr (G.N.); 2 Department of Radiation Oncology, Centre Jean Perrin, 58, rue Montalembert, 63011 Clermont-Ferrand cedex, France; E-Mail: celine.benoit@cjp.fr; 3 Laboratory of Biostatistics, Faculté de médecine, 4, rue Kirschleger, 67085 Strasbourg cedex, France; E-Mail: francois.lefebvre@chru-strasbourg.fr

**Keywords:** brain metastases, breast cancer, triple negative subtype, prognostic factors

## Abstract

To evaluate the prognostic factors and indexes of a series of 93 patients with breast cancer and brain metastases (BM) in a single institution. Treatment outcomes were evaluated according to the major prognostic indexes (RPA, BSBM, GPA scores) and breast cancer subtypes. Independent prognostic factors for overall survival (OS) were identified. The median OS values according to GPA 0–1, 1.5–2, 2.5–3 and 3.5–4, were 4.5, 9.5, 14.2 and 19.1 months, respectively (*p* < 0.0001) and according to genetic subtypes, they were 5, 14.2, 16.5 and 17.1 months for basal-like, luminal A and B and HER, respectively (*p* = 0.04). Using multivariate analysis, we established a new grading system using the six factors that were identified as indicators of longer survival: age under 60 (*p* = 0.001), high KPS (*p* = 0.007), primary tumor control (*p* = 0.05), low number of extracranial metastases and BM (*p* = 0.01 and 0.0002, respectively) and triple negative subtype (*p* = 0.002). Three groups with significantly different median survival times were identified: 4.1, 9.5 and 26.3 months, respectively (*p* < 0.0001). Our new grading system shows that prognostic indexes could be improved by using more levels of classification and confirms the strength of biological prognostic factors.

## 1. Introduction

Breast cancer has the second highest rate of brain metastases (BM) (30%) after lung cancer (34%) [1–4]. The optimal therapy for BM patients remains a controversial subject. Whole brain radiation therapy (WBRT) has been a leading treatment option for years; however, due to the development of new treatment modalities such as radiosurgery, discussing the most effective method to treat a particular patient has become a major focus. The prognosis of patients with BM remains poor, with a median survival less than one year after the diagnosis of BM [5]. Predictive factors for BM with breast cancer have been identified, such as an overexpression of human epidermal growth factor receptor 2 (HER2), a lack of expression of hormone receptors, a patient age under 50 years, a triple negative subtype and the presence of lung metastases [6–10]. The incidence of BM is particularly high in HER2-overexpressing breast cancer both due to the increased incidence of extracranial metastases, as well as to the effect of HER2+ tumor cells, which may have a marked brain tropism [11,12]. The outcome of patients with breast cancer varies with the genetic subtype. Patients with basal-like breast cancer (triple negative, HER2/ER/PR-negative) have a worse prognosis than those with the luminal A (HER2-negative, ER/PR-positive), luminal B (triple positive, HER2/ER/PR-positive) or HER2 (HER2-positive, ER/PR-negative) subtypes [13–15]. Several prognostic indexes were identified previously. Prognostic indexes are used to predict survival periods of patients with BM to determine if a patient would benefit from therapy before treatment begins. The Radiation Oncology Therapy Group (RTOG) established prognostic factors using Recursive Partitioning Analysis (RPA) in 1997, which were then re-evaluated and updated in the form of the Graded Prognostic Assessment (GPA) [16,17]. The GPA is a validated prognostic index for patients with BM and was deduced from an analysis of 1960 patients in the RTOG database. The GPA uses four criteria (age, Karnofsky performance status (KPS), number of BM, and whether extracranial metastases are present or absent), and it gives each factor a score of 0, 0.5, or 1.0. The patient with the best prognosis would have a GPA of 4.0. The prognostic factors vary by diagnosis, and several GPA indexes have been determined for several histological tumor types, including breast cancer. The array of prognoses created using this method can be used to guide therapy. For example, it can be used to avoid over-treatment of patients who have a very poor prognosis and to avoid under-treatment of patients with a favorable prognosis. The purpose of this study was to evaluate the outcomes of 93 patients with BM from breast cancer and identify diagnosis-specific prognostic factors.

## 2. Methods and Materials

### 2.1. Study Design and Patient Population

The present study was a single institutional retrospective analysis of a database of 93 patients with breast cancer treated for BM between September 2005 and December 2010 at the Center Paul Strauss, France. Diagnosis was established through contrast-enhanced cerebral computed tomography (CT) (55.9%), magnetic resonance imaging (MRI) (14%) or both (30.1%). BM were supratentorial (30.1%), subtentorial (8.6%) or both (60.2%). The BM site of one patient (1.1%) was unknown. Patients with a median age at diagnosis of 57 years old (27–83.1) were treated with surgery followed by WBRT or with WBRT alone in 15% and 85% of cases, respectively. Twenty-nine patients with one or two BM (31.2%) received a radiation boost at the operative site, and 64 patients (68.8%) with one or two BM did not receive a boost. All patients underwent a CT-scan for delineation, and a customized plastic mask was made to improve the set-up. The median interval between BM diagnosis and WBRT was 39 days (1–1005 days). WBRT was performed on all patients with 6 MV photons from a linear accelerator using bilateral fields. The radiation boost at the operative site was performed in some patients with one or two BM. It was delivered at 6 or 25 MV, using two or three fields after 3-dimensional treatment planning. Patients were treated according to their RPA class. Patients in RPA class I treated with radiation therapy alone received a WBRT at 37.5 Gy in 15 fractions of 2.5 Gy (five fractions per week), with a radiation boost of 10 Gy in four fractions of 2.5 Gy. Patients in RPA class I treated with surgical resection followed with radiation therapy received a WBRT of 40 Gy in 20 fractions of 2 Gy with a radiation boost of 16 Gy in eight fractions of 2 Gy (five fractions per week). Patients in RPA class II received a WBRT of 30 Gy in 10 fractions of 3 Gy (five fractions per week) with a radiation boost of 9 Gy in three fractions of 3 Gy, and patients in RPA class III received a WBRT at 20 Gy in five fractions of 4 Gy and one week. The median dose of irradiation was 36 Gy (4–56 Gy) in fractions of 2 to 4 Gy. The median boost dose was 11.3 Gy (5–16 Gy) in 2 to 3 Gy per fraction.

The RPA, BSBM (Basic score for brain metastases) and GPA scores were calculated for all patients [16–19]. The patient characteristics are listed in Table 1.

### 2.2. Statistical Analysis

The primary endpoint for these analyses was the overall survival (OS) from the last day of radiotherapy. These OS data were compared to the calculated OS determined through the identification of prognostic factors. All patients alive at the time of the analysis were censored according to the date of their last follow-up. The prognostic factors for survival time were determined. Survival probabilities were calculated using the Kaplan-Meier method for the subgroups defined by genetic subtype, RPA, GPA, BSBM and KPS. The log-rank test was used to evaluate whether significant survival differences were present among the different groups. Multivariate survival analyses were performed using the Cox proportional hazards model. Seventeen potential prognostic factors were evaluated with respect to the overall survival: age, KPS, control of primary tumor, presence of extracranial metastases (ECM), number of ECM and BM, chemotherapy at diagnosis of BM, presence of neurologic symptoms, localization of BM, site of BM, presence of estrogen (ER) and progesterone receptors (PR), overexpression of human epidermal growth factor receptor 2 (HER 2), proliferation index KI67, tumor grade, anatomopathology of tumor, and genetic subtype, including the triple negative subtype. All *p* values < 0.05 were considered statistically significant. The observed survival curves were compared using the curves derived through the log-rank test.

## 3. Results

Two patients were men. The tumor genetic subtypes were basal-like in 24 cases (25.8%), luminal A in 21 cases (22.6%), luminal B in 23 cases (24.7%), HER in 17 cases (18.3%) and data were not available in eight cases (8.6%). Patients were RPA I, II and III in 12.9%, 73.1%, and 14% of cases, respectively, GPA 0–1, 1.5–2, 2.5–3, 3.5–4 and unknown in 12.9%, 23.7%, 26.9%, 24.7% and 11.8% of cases, respectively, and BSBM 0, 1, 2, 3 and unknown in 7.5%, 38.7%, 43%, 7.5% and 3.3% of cases, respectively. Patients had KPS 90–100, 70–80 and <70 in 27 (29%), 51 (54.8%) and 12 cases (12.9%), respectively. Forty patients (43%) had one to three BM, and 53 patients (57%) had more than three BM. Seventy-seven patients had extracranial metastases (82.8%), 31 patients (40.3%) had one ECM and 46 patients (59.7%) had two or more ECM. The primary tumor was controlled for 73 patients (78.5%) at the time of BM diagnosis. Neurological symptoms were observable at the time of BM diagnosis for 66 patients (71%). Sixty patients received chemotherapy (64.5%) at time of BM diagnosis. The median interval between BM diagnosis and WBRT was 29 days (0–461 days).

### 3.1. Median Survival by RPA Class, BSBM, GPA Score, KPS and Diagnosis

The overall patients’ median follow-up was 10.4 months (0.1–72.7 months). The median follow-up of surviving patients was 37.3 months (17–72.7 months). Fifteen patients (16.1%) were still alive at the analysis, and 78 (83.9%) had died. Of those who had died, 24 patients (30.8%) died of neurologic symptoms, and 28 patients (35.9%) died from other events. We did not know the cause of death for 33.3% of patients.

The median OS values after treatment for RPA I, II and III were 37.3, 10.2, 3.5 months, respectively (*p* < 0.0001), and the median OS for GPA 0–1, 1.5–2, 2.5–3 and 3.5–4 were 4.5, 9.5, 14.2 and 19.1 months, respectively (*p* < 0.0001). The median OS values for BSBM 0, 1, 2 and 3 were 6.2, 5.2, 19.1 and 33.4 months, respectively (*p* < 0.0001), and according to KPS the median OS values were 19.4 months for KPS 90–100, 7.2 for KPS 70–80 and 4.5 for KPS < 70 (*p* = 0.0006). In the univariate analysis, the GPA, RPA and BSBM scores and the KPS were significantly associated with survival. The 6-, 12-, 18- and 24-month OS values according to prognostic indexes are shown in Table 2. The median OS values for pathologic subtypes for basal-like, HER, luminal B and luminal A were 5.0, 17.1, 16.5 and 14.2 months, respectively (*p* = 0.2). Patients with the basal-like subtype had significantly worse outcomes than all other patients, with OS values of 5.0 and 16.5 months for basal-like subtype and all other patients, respectively (*p* = 0.04). The 6-, 12-, 18- and 24-month OS values according to genetic subtypes are summarized in Table 3.

Univariate analysis of diagnosis-specific prognostic factors.

In univariate analysis, OS was associated with age (*p* = 0.02), KPS (*p* = 0.0004), control of primary tumor (*p* = 0.02), presence of ECM (*p* = 0.05), number of ECM (*p* = 0.0004), number of BM (*p* = 0.002), localization of BM (*p* = 0.002), anatomopathology (*p* = 0.002), presence of progesterone receptor (*p* = 0.01) and triple negative subtype (*p* = 0.04). The other factors tested were not significant.

Multivariate analysis of diagnosis-specific prognostic factors with favorable independent prognostic factors for OS in multivariate analysis were a patient age under 60 years (HR 3.83 CI 95% 1.68–8.71, *p* = 0.001), a high KPS (HR 3.17 CI 95% 1.35–7.47, *p* = 0.007), a number of ECM < 2 (HR 2.7 CI 95% 1.24–5.86, *p* = 0.01), a number of BM ≤ 3 (HR 3.5 CI 95% 1.8–7.02, *p* = 0.0002) and control of the primary tumor (HR 0.52 CI 95% 0.27–1.00, *p* = 0.05). The presence of the triple negative subtype was an unfavorable prognostic factor (HR 2.48 CI 95% 1.36–4.53, *p* = 0.002).

### 3.2. Local GPA

**Figure 1:**
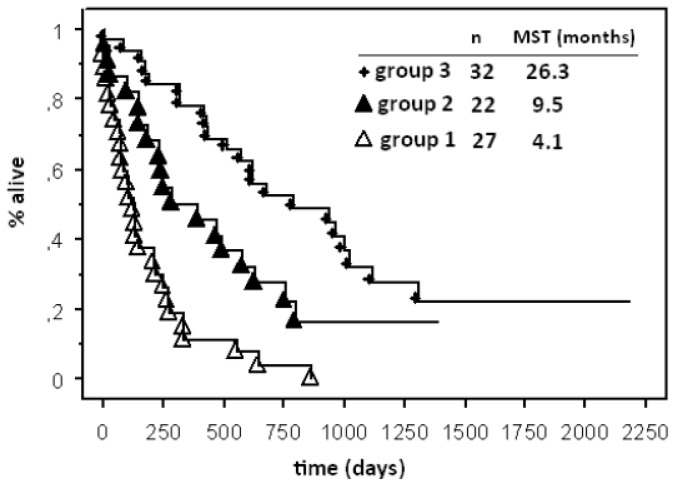
Kaplan-Meier curves for overall survival for our new local grading system.

We created an index in which each factor mentioned above was scored (0, 0.5, or 1), and these scores were then summed to yield a single number. Scores 0 or 1 were attributed to factors that were the most significant, as well as the age, the triple negative subtype, the number of BM and ECM. Because the control of the primary tumor was the worst subjective and less significant factor, it was given a score of 0 or 0.5 only. Scores 0, 0.5, or 1 were attributed for KPS < 70, KPS 70–80 and KPS >90, respectively. Our index sums the score of the six items. Our new grading system is shown in Table 4. We were able to identify three groups with significantly different median survival times. Group 1 represented patients with scores from 0.5 to 2.5, Group 2 included patients with scores from 3.0 to 3.5 and Group 3 included patients with scores from 4.0 to 5.5. The numbers of patients in each group were 27, 22 and 32 in groups 1, 2 and 3, respectively. The first group had the worst outcome, with a median OS of 4.1 months, and the second group exhibited a median OS of 9.5 months. The patients in the third group had the best outcome, with a median OS of 26.3 months, (*p* < 0.0001) (Figure 1). In our analysis, the three groups defined by the score were distinct from one another at a worst level of significance of *p* = 0.04.

## 4. Discussion

Our study is a retrospective analysis of the outcomes of 93 patients treated at a single institution with homogeneous therapies. Median OS values for RPA classes I, II and III were 37.3 10.2 and 3.5 months, respectively (*p* < 0.0001). In our series, patients had better prognostic outcomes according to RPA than those in the RTOG database [17]. Although this score was established with a cohort of patients with different types of primitive tumors, it does not consider the variable outcomes of these patients. In this study, only 12% of patients had BM with breast cancer, and no parameter specific for breast cancer was analysed. The median OS values according to the BSBM score also differ between patients in our studies and those of the series reported by Lorenzoni *et al.*[19]. For BSBM 0, 1, 2 and 3, the OS values were 6.2, 5.2, 19.1 and 33.4 months in our study, and they were 2.2, 3.4, 5.1 and 7.0 months in the Lorenzoni *et al.* study, respectively. The RPA and BSBM scores have three prognostic factors in common (KPS, control of primary tumor and presence of extracranial metastases) and exhibit similar prognostic capabilities. Neither the RPA nor the BSBM considers the number of metastases. Additionally, the RTOG 9508 demonstrated a better outcome for patients with one metastasis compared to those with two or three BM [20]. Moreover, they are based on factors that are rather subjective; in fact, the evaluation of parameters such as the control of primary tumor or systemic disease status remains uncertain because it varies according to the type, technique, and timing of the restaging studies. For example, the control of the primary tumor is a commonly observed factor in breast cancer but not in lung cancer. In 2008, Sperduto *et al.*[16] established the GPA score, which was the first score to consider the type of primitive tumor. This prognostic index is the least subjective and most quantitative of all previous indexes. For breast cancer, only KPS has been shown to be a significant independent prognostic factor; however, Sperduto has recently established the breast GPA score after a multi-institutional retrospective analysis of 400 breast cancers treated for newly diagnosed BM [18]. This score is the first to add parameters that were not present in the other scores examined thus far. The breast cancer GPA is based on four factors: age, KPS, Human Epidermal Growth Factor Receptor (HER2), estrogen and progesterone receptor (ER/PR) status. The genetic subtypes are basal (triple negative, HER 2/ER/PR-negative), Luminal A (HER2-negative, ER/PR-positive), Luminal B (triple positive, HER2/ER/PR-positive), and HER2 (HER2-positive, ER/PR-negative). The breast GPA score is the sum of the scores for each of the three factors (KPS, genetic subtype and age). Each is given a score of 0 to 2.0, as follows: 0.0 (KPS < 60, basal, age > 60), 0.5 (KPS 60, age < 60), 1.0 (KPS 70–80, luminal A), 1.5 (KPS 90–100, HER2), 2.0 (luminal B). Through this method, the Breast GPA score has improved its accuracy for predicting survival in patients with BMs. The median OS values for GPA scores 0–1, 1.5–2, 2.5–3 and 3.5–4 were 3.4, 7.7, 15.1 and 25.3 months, respectively (*p* < 0.0001). Our findings are in accordance with data regarding the OS of patients with BM presented by Sperduto *et al.*[18], and they exhibit a dramatic range of survival for women treated for BM with breast cancer (4.5–19.1 months). This score is significantly more accurate in predicting the outcome compared to the RPA score. For example, the same patient could be RPA class II with a predicted outcome of 10.2 months according to our study and have a GPA class 4 with a predicted outcome of 19.1 months. This discrepancy could have many consequences for decisions made during treatment. Patients who survive for a long period of time after treatment are exposed to an increased risk of long-term complication, particularly a decline in neurocognitive function after WBRT. Therefore, therapies must be adapted, and other treatment modalities such as surgery or radiosurgery, alone or combined, can be discussed. Another prognostic index that included a biologic prognostic factor was established by Le Scodan *et al.*[21]. They retrospectively analyzed the outcomes of 117 patients with BM from breast carcinoma treated with WBRT using multivariate analysis. RPA class III, lymphopenia and hormone receptor negative status were independent prognostic factors for poor survival.

In our series, six factors were identified as impacting long-term survival by means of multivariate analysis. Our results confirm the significant prognostic factors used by the Breast GPA score established by Sperduto *et al.*[18]. In the study by Sperduto *et al.*, improved OS was significantly associated with age, KPS and triple negative subtype on multivariate analysis. However, some differences between our study and the study by Sperduto et al. have been identified. Control of the primary tumor and the numbers of ECM and BM were also significant prognostic factors that could be added to age, KPS and genetic subtype.

Our study presents several limitations of the design. It is a retrospective series and has a sample size, consequently these results should be interpreted with caution. Control of the primary tumor is one factor that has less significance and is less objective, but it is almost always achieved in the case of breast cancer: for 78.5% of patients in our series, the primary tumor was controlled, and a local relapse is easier diagnosable in breast than in lung cancer, mainly for example after local radiation treatment. Another weakness could be the number of extracranial metastases sites. However, because the patients are treated in the same area, and because physicians follow equivalent guidelines for state patients, this data is probably more relevant than in other series where the number of referring areas is more numerous. The last factor of number of BM is always disputable because patients with less than three BM did not have a systematic MRI to confirm the number of BM. However, our new local grading system shows that prognostic indexes could be improved with more possible levels. These levels could be useful in making decision for patients with BM. However the strength of the prognostic factors needs to be confirmed with a larger database. Furthermore, today, because of the large database used for patients to determine the breast GPA score, this remains as the reference for proposal of the best treatment of BM from breast cancer.

## 5. Conclusions

Several scores can be used to predict the survival periods of patients with brain metastases from breast cancer after various treatment options. Our series confirms the strength of prognostic factors used in the Breast GPA score (age, KPS, genetic subtype). In our single institutional series, the control of the primary tumor and the number of ECM and BM were significant prognostic factors also. Of all the indexes used, the Breast GPA score is the least subjective and most quantitative. Including the genetic subtype in the new breast GPA score has improved its sensitivity and showed that the inclusion of other biologic prognostic factors might improve results, could be important for clinical application, and should be considered in the management of patients with brain metastases.

## Figures and Tables

**Table 1:** Patient characteristics.

Characteristic	Value
Age (y)	
Median	57 (27–83.1)
Gender (*n*)	
Male	2 (2.1)
Female	91 (97.9)
KPS (*n*)	
<70	12 (12.9)
70–80	51 (54.8)
90–100	27 (29)
Unknown	3 (3.3)
BM (*n*)	
1–3	40 (43)
>3	53 (57)
ECM (*n*)	
Yes	77 (82.8)
No	16 (17.2)
Unknown	
Number of ECM (*n*)	
1	31 (40.3)
≥2	46 (59.7)
Control of primary tumor (*n*)	
Yes	73 (78.5)
No	20 (21.5)
Neurological symptoms (*n*)	
Yes	66 (71)
No	27 (29)
Chemotherapy (*n*)	
Yes	60 (64.5)
No	33 (35.5)
Surgical resection (*n*)	
Yes	14 (15)
No	79 (85)
RPA (*n*)	
Class I	12 (12.9)
Class II	68 (73.1)
Class III	13 (14)
GPA (*n*)	
0–1	12 (12.9)
1.5–2	22 (23.7)
2.5–3	25 (26.9)
3.5–4	23 (24.7)
Unknown	11 (11.8)
BSBM (*n*)	
0	7 (7.5)
1	36 (38.7)
2	40 (43)
3	7 (7.5)
Unknown	3 (3.3)
Genetic subtype (*n*)	
Basal	24 (25.8)
HER	17 (18.3)
Luminal A	21 (22.6)
Luminal B	23 (24.7)
Unknown	8 (8.6)

KPS, Karnofsky performance status; BM, brain metastases; ECM, extracranial metastases; RPA, recursive partitioning analysis; GPA, graded prognostic assessment; BSBM, basic score for brain metastases.

**Table 2:** Univariate analysis evaluating the RPA class, GPA, BSBM and KPS for overall survival.

Variable	OS at 6 months (%)	OS at 1 year (%)	OS at 1 1/2 year (%)	OS at 2 years (%)	*p* (log-rank test)
RPA					<0.0001
Class I	91	83.3	77	74.1	
Class II	64.7	45	33.8	25	
Class III	33.3	8.3	8.3	-	

GPA					<0.0001
0–1	15	0	-	-	
1.5–2	68.4	40.5	36.4	28	
2.5–3	64	50	42	30.8	
3.5–4	82.6	70	49	34.5	

BSBM					<0.0001
0	42.9	14.3	-	-	
1	41	21	8	5.7	
2	75	60	53	40	
3	100	100	100	83.3	

KPS					=0.0006
90–100	90	73	55.1	43.3	
70–80	52.9	37	30	22	
<70	36.4	9.1	9.1	-	

OS, overall survival; RPA, recursive partitioning analysis; GPA, graded prognostic assessment; BSBM, basic score for brain metastasis; KPS, Karnofsky performance status.

**Table 3:** Overall survivals by diagnosis.

Diagnosis	OS at 6 months (%)	OS at 1 year (%)	OS at 1 1/2 year (%)	OS at 2 years (%)	*P* (log-rank test)
Basal	42	20	17.4	13	=0.04
HER	70	59	45	23	
Luminal A	75	50	44	38.1	
Luminal B	75	58	45	38.3	
unknown	25	-	-	-	
Total	64	45	37	29.1	

OS, overall survival.

**Table 4:** Our new grading system.

Scores	0	0.5		1.0
Age (year)	≥60	70–80		<60
KPS	<70			90–100
Basal subtype	yes			no
Number of ECM≥2	yes			no
Number of BM>3	yes			no
Control of primary Tumor	no	yes		
**Scores**	**0–2.5**	**3.0–3.5**	**4.0–5.5**	
Local GPA	1	2	3	

KPS, Karnofsky performance status; BM, brain metastases; ECM, extracranial metastases; RPA, recursive partitioning analysis; GPA, graded prognostic assessment; BSBM, basic score for brain metastases.
